# Role of inflammasomes in acute respiratory distress syndrome

**DOI:** 10.1136/thorax-2024-222596

**Published:** 2025-01-30

**Authors:** Luke Flower, Emilio G Vozza, Clare E Bryant, Charlotte Summers

**Affiliations:** 1 Victor Phillip Dahdaleh Heart & Lung Research Institute, University of Cambridge, Cambridge, UK; 2 Cambridge University Hospitals NHS Foundation Trust, Cambridge, UK

**Keywords:** ARDS, Neutrophil Biology, Innate Immunity

## Abstract

Acute respiratory distress syndrome (ARDS) is present in >10% of all people admitted to critical care and is associated with severe morbidity and mortality. Despite more than half a century since its first description, no efficacious pharmacological therapies have been developed, and little progress has been made in improving clinical outcomes. Neutrophils are the principal drivers of ARDS, with their priming and subsequent aberrant downstream functions, including interleukin (IL) 1β and IL-18 secretion, central to the disease pathogenesis. The dominant pathways through which IL-1β and IL-18 are believed to be elaborated are multimeric protein structures called inflammasomes that consist of sensor proteins, adaptor proteins and an effector enzyme. The inflammasome’s initial activation depends on one of a variety of damage-associated (DAMP) or pathogen-associated (PAMP) molecular patterns. However, once activated, a common downstream inflammatory pathway is initiated regardless of the specific DAMP or PAMP involved. Several inflammasomes exist in humans. The nucleotide-binding domain leucine-rich repeat (NLR) family, pyrin domain-containing 3 (NLRP3), inflammasome is the best described in the context of ARDS and is known to be activated in both infective and sterile cases. The NLR family, caspase activation and recruitment domain-containing 4 (NLRC4) and absent in melanoma 2 (AIM2) inflammasomes have also been implicated in various ARDS settings, as have inflammasome-independent pathways. Further work is required to understand human biology as much of our knowledge is extrapolated from rodent experimental models. Experimental lung injury models have demonstrated beneficial responses to inflammasome, IL-1β and IL-18 blockade. However, findings have yet to be successfully translated into humans with ARDS, likely due to an underappreciation of the central role of the neutrophil inflammasome. A thorough understanding of inflammasome pathways is vital for critical care clinicians and researchers and for the development of beneficial therapies. In this review, we describe the central role of the inflammasome in the development of ARDS and its potential for immunomodulation, highlighting key areas for future research.

## Introduction

 Acute respiratory distress syndrome (ARDS) describes a clinical syndrome of aberrant pulmonary inflammation, non-cardiogenic pulmonary oedema and severe hypoxaemia and is associated with significant morbidity and mortality.^1 2^ Despite more than half a century passing since its first description, no effective pharmacological therapies have been developed and minimal progress has been made in improving patient outcomes.^2^,^4^

The innate immune system plays a central role in ARDS through neutrophil priming, secretion of inflammatory mediators and activation of downstream inflammatory pathways.^1 5 6^ Elevated concentrations of pro-inflammatory mediators, including interleukin (IL) 1β and IL-18, are associated with worsened outcomes in ARDS, and blockade is beneficial in experimental lung injury models.^7^,^10^ These inflammatory mediators are predominantly processed via a multimeric protein structure called the inflammasome, which consists of sensor proteins, adaptor proteins and an effector enzyme.^11^ The inflammasome pathways used in some immune cell types (eg, macrophages) are well-characterised, but there remains a paucity of research in human neutrophils despite their central role in the pathogenesis of acute inflammatory conditions such as ARDS.^12^,^14^ We describe the role of the inflammasome in the pathogenesis of ARDS and its potential for immunomodulation and highlight areas for future research.

### Acute respiratory distress syndrome

The Berlin Criteria are the most widely accepted definition of ARDS, describing it as severe hypoxaemia (a PaO_2_/FiO_2_ ratio of <39.9 kPa) necessitating a positive end-expiratory pressure of ≥5 cmH_2_O, developing within 1 week of the presumed insult, in association with bilateral infiltrates on chest radiography not fully explained by cardiogenic pulmonary oedema.^15^

ARDS has been categorised into subgroups by the degree of inflammatory response observed, namely: ‘hyper-inflammatory’ and ‘hypo-inflammatory’.^16^ The hyperinflammatory subgroup accounts for approximately 30% of cases and is associated with increased mortality, excess inflammasome activation and secretion of the pro-inflammatory cytokines IL-1β and IL-18.^8 16 17^

**Figure 1:**
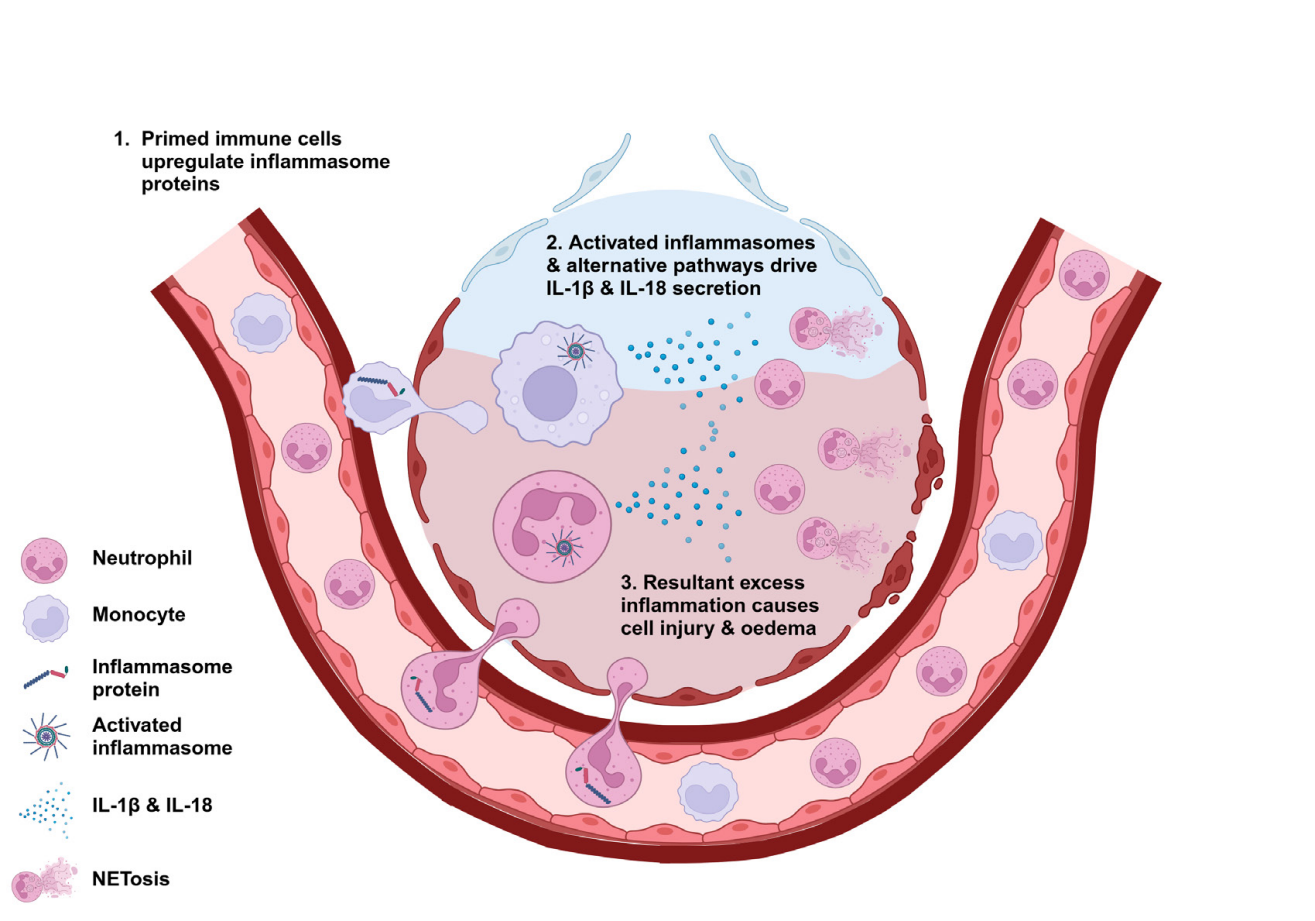
A summary of the role played by inflammasomes in ARDS pathophysiology. (1) In ARDS, circulating immune cells are activated, and inflammasome priming occurs, which leads to the upregulation of inflammasome proteins. These activated immune cells become trapped in pulmonary capillaries and migrate into the alveoli. (2) Once in the alveoli, activation of inflammasome-dependent and inflammasome-independent pathways leads to the secretion of pro-inflammatory cytokines, including IL-1β and IL-18, resulting in a dysregulated and injurious inflammatory response (ie, excess reactive oxygen species production and neutrophil extracellular trap formation NETosis). (3) In ARDS, this excess inflammatory response leads to tissue damage, disruption of the alveolar-capillary membrane, pulmonary oedema and resultant hypoxaemia, morbidity and mortality. ARDS, acute respiratory distress syndrome; IL, interleukin.

Neutrophils are one of the principal protagonists of the aberrant inflammatory response seen in ARDS.^1 18 19^ On exposure to pro-inflammatory stimuli (eg, infection, trauma) neutrophils become primed and migrate into the alveoli. Once there, inflammasome activation contributes to pro-inflammatory cytokine secretion (eg, IL-1β and IL-18) and downstream inflammatory pathways, including neutrophil extracellular trap release (NETosis). These responses lead to tissue damage, alveolar-capillary membrane disruption, alveolar oedema and, ultimately, refractory hypoxaemia (figure 1).^1 10 19^ Therefore, immunomodulation of inflammasomes and downstream aberrant neutrophil responses represent a promising avenue for ARDS therapies.^17 20^

### Inflammasomes

Inflammasomes are multimeric protein structures that, on sensing an activation signal, initiate a common downstream pathway that results in IL-1β and IL-18 secretion and an inflammatory form of cell death termed pyroptosis.^11^ While beneficial in the initial response to infection, inflammasome dysfunction has been shown to contribute to multiple disease states, including ARDS.^10 21^ Our current understanding of inflammasomes is primarily built on data obtained from human macrophage and animal studies, with a detailed characterisation of their role in human neutrophils representing an unmet scientific need.^22^,^24^

Inflammasomes comprise three main components: a pattern recognition receptor (PRR) that acts as the sensor protein, an adaptor protein and an effector enzyme. The PRR may hold one of several structures, with the key inflammasomes identified in human disease including NLRP1 (nucleotide-binding domain leucine-rich repeat (NLR) family, pyrin domain (PYD)-containing 1), NLRP3 (NLR, PYD-containing 3), NLR family apoptosis inhibitory protein (NAIP)/NLRC4 (NLR family, caspase activation and recruitment domain (CARD)-containing 4), absent in melanoma 2 (AIM2), pyrin and CARD8. A non-canonical pathway also exists whereby caspase-4 and caspase-5 directly recognise cytoplasmic lipopolysaccharide (LPS), leading to gasdermin-D (GSDMD) cleavage and cell membrane pore formation. This membrane disruption allows for the release of inflammatory cytokines, trans-membrane ion flux and the release of damage-associated molecular patterns (DAMPs), which can activate the NLRP3 inflammasome.^11^ Table 1 provides an overview of the primary inflammasomes in humans, although some uncertainty remains regarding the specific cell types in which they are present.

**Table 1:** Known key inflammasomes found in humans, their activators and cell types where they have been reported to exist

Inflammasome	Known activators	Known human cell type where present	References
NLRP1	Bacterial toxins (eg, *Bacillus anthracis lethal toxin),* viral dsRNA, viral proteases, intracellular ATP depletion, muramyl dipeptide, N-terminal degradation(eg, SARS-CoV-2 3CL), DPP8/9 inhibition, ultraviolet A and B	Neutrophils, monocytes- macrophages, dendritic cells, airway epithelial cells, keratinocytes	110 114
NLRP3	Extracellular ATP, mitochondrial DNA, amyloid-β prion protein, cholesterol and monosodium urate crystals, alum, silica, imiquimod, ion flux (eg, K^+^ efflux, Ca^2+^ influx, Cl^−^ efflux), lysosomal proteases, ROS, aluminium salts, hyperosmotic stress, metabolic shifts, LPS, peptidoglycan, pore-forming ionophores (eg, nigericin), P2X7R, gramicidin, valinomycin, β-Glucan, dsRNA, ssRNA, ultraviolet A and B	Neutrophils, monocytes-macrophages, dendritic cells, oligodendrocytes, astrocytes, endothelial cells, retinal pigmented cells, epithelial cells	13 25 26 36 45 46 60 72 79 96 111 115
NAIP/NLRC4	Bacterial flagellin (eg, *Salmonella typhimurium*), rod and needle proteins from type III secretion systems (eg, PrgI from *Salmonella* spp, EspB and EspD from *Escherichia coli*), hyperosmotic stress, unidentified metabolic drivers	Neutrophils, monocytes-macrophages, dendritic cells, airway epithelial cells, intestinal epithelial cells, astrocytes	34 51 65 68 81
PYRIN	Actin skeleton disruption, RhoA-GTPase inactivation (eg, TcdB from *Clostridium difficile*)	Monocyte-macrophages, dendritic cells	111 116 117
AIM2	Cystolic dsDNA from viruses, bacteria (eg, *Mycobacterium tuberculosis*), fungi, autoimmune disease or tissue damage and associated cell death (eg, traumatic brain injury or burns)	Neutrophils, monocytes-macrophages, dendritic cells, keratinocytes, T regulatory cells	22 67 82 111 118
CARD8	Inhibition of DPP8/9, viral proteases (eg, HIV-1^PR^, Picornavirus, SARS-CoV-2)	Monocytes-macrophages, T cells	111 119 120

AIM, absent in melanoma; ATP, adenosine triphosphate; dsDNA, double-stranded DNA; dsRNA, double-stranded RNA; LPS, lipopolysaccharide; NLRC, NLR family, caspase activation and recruitment domain (CARD)-containing; NLRP, nucleotide-binding domain leucine-rich repeat (NLR) family, pyrin domain (PYD)-containing; ROS, reactive oxygen species.

**Figure 2:**
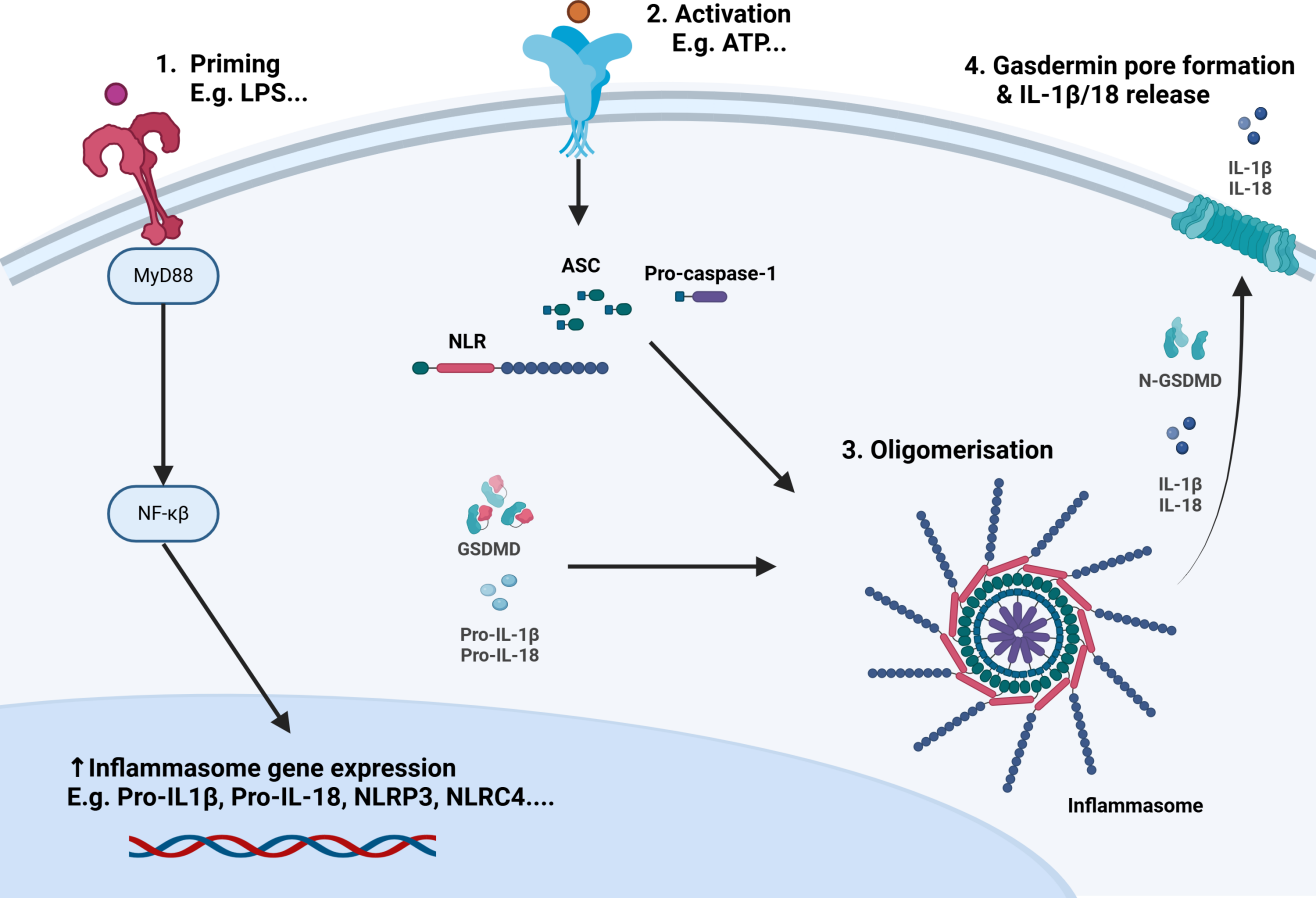
A summary of the canonical inflammasome pathway. The first step in the canonical inflammasome pathway requires a priming signal (damage-associated molecular pattern or pathogen-associated molecular pattern) that interacts with membrane-bound receptors such as Toll-like receptor 4. Downstream of this, myeloid differentiation primary response protein (MyD88) and nuclear factor kappa-light-chain-enhancer of activated B cells (NF-κB) are activated, leading to transcriptional upregulation of the relevant NLR protein, pro-IL-1β and pro-IL-18. A second activation signal, such as pathogens, particulate matter or extracellular ATP, is required for the inflammasome complex to oligomerise. After oligomerisation, the apoptosis-associated speck-like protein (ASC) attaches to the structure, forming an ASC speck to which pro-caspase-1 binds and then undergoes proximity-induced autoproteolytic cleavage to its active p33/p10 form. Caspase-1 then cleaves pro-IL1β and pro-IL-18 to their active forms, and gasdermin-D (GSDMD) is cleaved to N-GSDMD, which translocates to the cell membrane to form pores that facilitate release of inflammatory mediators and pyroptosis. ATP, adenosine triphosphate; IL, interleukin; LPS, lipopolysaccharide; NLRC, NLR family, caspase activation and recruitment domain (CARD)-containing; NLRP, nucleotide-binding domain leucine-rich repeat (NLR) family, pyrin domain (PYD)-containing.

The most studied inflammasome is NLRP3, which is unique in the broad range of DAMPs and pathogen-associated molecular patterns (PAMPs) it can detect.^25^,^27^ Common downstream mechanisms have been described through which NLRP3 detects diverse stimuli (eg, ion flux, ROS production, or endosomal disruption).^28^ The first step in the canonical NLRP3 inflammasome pathway, which is shared by other inflammasomes (eg, NAIP/NLRC4), requires a priming signal that may be a DAMP or PAMP (eg, adenosine-tri-phosphate (ATP) and LPS, respectively). This priming signal interacts with membrane-bound receptors such as Toll-like receptor 4 (TLR4), leading to transcriptional upregulation of the relevant NLR-, pro-IL-1β and pro-IL-18. NLRP3 then undergoes post-translational modifications that are key to its activation, including deubiquitination of its leucine-rich repeat domain to enable oligomerisation, phosphorylation and sumoylation.^11 25^ A second activation signal (eg, extracellular ATP) is then required, which stimulates inflammasome oligomerisation.^2529^,^31^ After oligomerisation, the adaptor protein apoptosis-associated speck-like protein containing a caspase recruitment domain (ASC) attaches to the complex, forming an ASC speck, to which pro-caspase-1 binds and undergoes proximity-induced autoproteolytic cleavage and activation.^32^ Caspase-1 then cleaves pro-IL1β and pro-IL-18 to their active forms and GSDMD to N-GSDMD, which forms pores in the cell membrane, facilitating the release of inflammatory mediators and pyroptosis (figure 2).^11 32^ While the upstream pathways differ between inflammasomes, the final outputs remain largely preserved.

#### Neutrophils

When examining the role of inflammasomes in ARDS, it is important to recognise that although neutrophils are key effectors of the innate immune system, most of our current understanding of inflammasomes is derived from studies involving human monocytes or animals.^22^,^2433^

However, significant differences in inflammasome expression exist between human neutrophils and other immune-cell subtypes and between mammalian species, limiting the validity of extrapolating such work into human disease.^22 34 35^ Bakele *et al* demonstrated that healthy human neutrophils primed with LPS express almost identical levels of IL-1β mRNA to primed peripheral blood monocytes, but lower levels of IL-18, NLRP3 and caspase-1 mRNA, and increased levels of NLRC4, AIM2 and ASC mRNA.^22^ Chen *et al* also demonstrated differing NLR inflammasome mRNA expression between neutrophils and monocytes in health.^34^

Human neutrophils show differences in inflammasome protein expression compared with monocytes and macrophages. For instance, detecting NLRP3 protein in neutrophils via methods like Western blotting is more challenging than in other cell types or for different inflammasomes.^22^ Nonetheless, human neutrophils have been shown to secrete IL-1β and form ASC specks when exposed to known NLRP3 activators.^36 37^ In mice, the presence of inflammasome-independent pathways in neutrophils is supported by the finding that caspase-1 is dispensable for the secretion of IL-1β during lung injury.^12^ These results have led to the search for alternative enzymes that cleave pro-IL-1β, with Franchi *et al* identifying a synergistic interaction between inflammasome-dependent and inflammasome-independent pathways that use neutrophil elastase, proteinase-3 and matrix metalloproteinase-9.^38 39^ Moreover, several groups have demonstrated serine proteases to be the predominant activators of IL-1β in animal models of sterile lung injury.^39 40^ These inflammasome-independent pathways have not yet been investigated in humans with ARDS.

Another cardinal feature of inflammasome activation that differs in human neutrophils is pyroptosis.^11^ Chen *et al* demonstrated that neither NLRC4 nor AIM2 activation induces pyroptosis in murine neutrophils, and Kovacs *et al* observed similar results following NLRC4 stimulation.^34 41^ One explanation for this relates to ASC speck size and the downstream effects on caspase-1 activity.^32^ Neutrophils form multiple small ASC specks rather than the single large specks seen in monocytes; as the intensity of the caspase-1 response is related to the size of the speck, lower intensity of caspase-1 activity in neutrophils, less GSDMD cleavage and insufficient pore formation to trigger pyroptosis occur.^32^ Another possible explanation is that inflammasome-independent pathways not involving GSDMD predominate in neutrophils.^40^ However, it should be noted that Chauhan *et al* demonstrate that murine neutrophils could undergo GSDMD-dependent pyroptosis in response to inflammasome agonists.^42^ The specifics of neutrophil pyroptosis remain an important unanswered scientific question.

Non-canonical inflammasome activation can trigger NETosis in human neutrophils, a non-pyroptotic mechanism of cell death. NETosis involves cell membrane disruption following activation of the non-canonical inflammasome and cleavage of GSDMD, resulting in the extrusion of granular components and chromatin.^43^ While this process has host defence properties, it can also contribute to tissue damage in ARDS.^44^

### Inflammasome in ARDS

Multiple inflammasomes have been implicated in the development of ARDS, with the specific pathway activated dependent on the inflammatory trigger (table 2).

**Table 2:** Inflammasomes implicated in ARDS development of varying aetiology

Aetiology of ARDS	Proposed inflammasome implicated	Species	Reference
Bacterial pneumonia	NLRP3	Human, mouse	46 47
	NLRC4	Human, mouse	14 51
Viral pneumonia	NLRP3	Human, mouse, rat	52 54121
Ventilation induced lung injury	Inflammasome-independent	Rat	12
	NLRP3	Mouse	58
Extra-pulmonary sepsis	NLRP3	Human, mouse	61 62
	NLRC4	Human, mouse	14 21 30 65
	AIM2 (protective)	Mouse	67
Pancreatitis	NLRP3	Mouse	69 70
	NLRC4	Mouse	68
Burns	NLRP3	Human, mouse, rat	72 73
	AIM2	Human, mouse	74
Traumatic brain injury	NLRP3	Human, mouse, rat	77 78 80 124
Ischaemia-reperfusion injury	NLRC4	Mouse, rat	83
	AIM2	Mouse	82
TRALI	NLRP3	Human	85

AIM, absent in melanoma; ARDS, acute respiratory distress syndrome; NLRC, NLR family, caspase activation and recruitment domain (CARD)-containing; NLRP, nucleotide-binding domain leucine-rich repeat (NLR) family, pyrin domain (PYD)-containing; TRALI, transfusion induced lung injury.

#### Infective pulmonary causes

Pneumonia is the leading cause of ARDS worldwide.^2 16^ Neutrophils are the predominant source of IL-1β in several causes of bacterial and viral pneumonia-induced lung injury, with inflammasome-dependent and inflammasome-independent mechanisms both implicated.^14 34^

In a murine model of *Streptococcus pneumonia* infection*,* secretion of the pore-forming toxin pneumolysin has been shown to stimulate the NLRP3 inflammasome, which initially has a protective response that becomes harmful as the disease progresses.^45^,^47^ Moreover, Hassane *et al* showed NLRP3-inflammasome dependent IL-1β secretion to be the principal regulator of the murine immune response to experimental *S. pneumonia* infection.^48^ The NLRP3 inflammasome has also been shown to be involved in experimental *Staphylococcus aureus* pneumonia in mice, where its activation and resultant pulmonary inflammation are believed to be harmful to the host.^49^ The NLRC4 inflammasome drives both *Pseudomonas aeruginosa* and isolated LPS-induced lung injury in mice, with NLRC4 knockout and inhibition of IL-1β both associated with reduced inflammation and improved outcomes.^14 50^ NLRC4 has also been implicated in *Legionella pneumonia*, *Burkholderia pseudomallei*, and *Klebsiella pneumonia* lung injury.^51^

Multiple viral causes of pneumonia have been shown to activate inflammasomes in humans, including SARS-CoV-1, SARS-CoV-2, Middle Eastern respiratory syndrome and influenza.^52^ Studies in humans showed neutrophil inflammasome activation secondary to SARS-CoV-2 infection, with increased ASC speck formation, IL-1β and IL-18 production, and pyroptosis, all associated with worsened disease severity.^53 54^ Similarly, work by both Leal *et al* and Aymonnier *et al* found the neutrophil inflammasome to be the predominant source of IL-1β in SARS-CoV-2-associated ARDS in humans.^55 56^ The human NLRP3 inflammasome also appears to be activated by influenza viruses in multiple immune and non-immune cells.^57^ Murine studies have further delineated the relationship between NLRP3, influenza A infection and lung injury, highlighting a protective effect in the early stages of the disease that becomes harmful as the disease progresses.^57^

#### Sterile pulmonary causes

Multiple non-infectious pulmonary causes result in ARDS, including aspiration of acidic gastric contents.^4^ Mizushina *et al* used IL-1β and NLRP3 knockout mice to demonstrate that NLRP3-independent secretion of IL-1β by neutrophils was the main driver of acid-induced lung injury and proposed a role for neutrophil elastase and proteinase-3.^40^ Mechanical ventilation can also cause sterile lung injury (ventilator-associated lung injury; VILI), whereby ventilation of inflamed lungs leads to a self-perpetuating cycle of aberrant inflammation and tissue damage.^12^ Increased inflammasome activation, IL-1β and IL-18 concentrations have been shown to correlate with VILI severity and their inhibition with improved outcomes in animal models.^24 58^ Timmermans *et al* used a rodent model to demonstrate that neutrophil serine proteases, rather than the NLRP3 inflammasome, are the predominant source of IL-1β maturation in VILI.^12^ A contrasting rodent study by Kuipers *et al* suggested that the NLRP3 inflammasome also contributed to VILI, demonstrating increased NLRP3 and ASC mRNA expression and caspase-1 activity, with NLRP3 and IL-1β inhibition both reducing the severity of murine lung injury.^58^ These conflicting findings may be explained by their respective ventilation strategies. Timmermans *et al* ventilated mice at 7.5 mL/kg, while NLRP3-dependent IL-1β secretion was only seen when Kuipers *et al* used 15 mL/kg volumes.^12 58^ A better understanding of these IL-1β secreting pathways in human neutrophils is required.

#### Extra-pulmonary infective causes

Aberrant inflammatory responses are also seen in patients with sepsis, where approximately 6% of patients progress to develop ARDS.^59^ The initial inflammasome activated will depend on the initiating pathogen, but a common final pathway is seen as the systemic inflammatory response develops.^59^ The inflammasome has been highlighted as a critical protagonist of this systemic inflammatory response in mice, with activation peaking at day 1, coinciding with peak serum IL-1β concentrations.^60^ NLRP3 is the most studied inflammasome in sepsis and may be stimulated by bacterial, viral and fungal infection alongside the various DAMPs released during sepsis (table 1).^61 62^ Huang *et al* found that increased NLRP3 expression is associated with worsening multi-organ failure in humans with sepsis, and Fukui *et al* demonstrated that the neutrophil NLRP3 inflammasome is the principal driver of early inflammation in murine experimental peritonitis.^61 62^ Inhibition of GSDMD and caspase-1 activation and blockade of NLRP3 and ASC upregulation have all been shown to protect against lung injury in a murine caecal ligation and puncture (CLP) model.^63^ This beneficial effect of NLRP3 blockade has been reproduced in studies investigating CLP and LPS-induced murine lung injury.^64^

While NLRP3 is the most studied inflammasome in sepsis-induced lung injury, alternative inflammasomes have been implicated.^21^ NLRC4 gene expression is raised in the innate immune cells of septic patients, the extent of which was associated with increasing mortality.^21^ The NLRC4 inflammasome is also implicated in the development of sepsis in humans in response to pathogens, including *Salmonella and P. aeruginosa.*^14 30^ Additionally, isolated upregulation of the NLRC4 inflammasome in neutrophils is sufficient to cause severe systemic inflammation, and Wang *et al* demonstrated that silencing of NLRC4 ameliorated lung injury and inflammation in murine CLP.^65 66^ In contrast, the AIM2 inflammasome is protective in the initial response to infection in mice, with their blockade associated with worsened outcomes.^67^

#### Extra-pulmonary sterile causes

ARDS may be triggered by sterile extra-pulmonary stimuli such as pancreatitis, major trauma, burns and massive transfusion of blood products, whereby stimulation of an excessive inflammatory response results in neutrophil activation and lung injury.^5^

In severe pancreatitis, self-digestion of pancreatic tissue leads to cellular damage, the release of DAMPS and inflammasome activation.^68 69^ IL-1β is transcribed by a range of cells in acute pancreatitis. Sendler *et al* demonstrated NLRP3 to be the principal driver of inflammation in a murine model of acute pancreatitis, with its inhibition associated with improved outcomes.^69^ Moreover, work from Yu *et al* identified the instrumental role of NLRP3 and nuclear factor kappa-light-chain-enhancer of activated B cells pathways in pancreatitis-induced lung injury in mice, with a reduction in lung inflammation and mortality seen following inhibition.^70^ The NLRC4 inflammasome has been less well-studied in models of acute pancreatitis, and its involvement in lung injury is yet to be defined.^68^

The tissue damage caused by severe burns results in the release of inflammasome agonists, with an estimated 35%–40% of mechanically ventilated patients with burns developing ARDS.^71^ Han *et al* showed that lung injury development following burns depended on ROS-mediated NLRP3 inflammasome activation.^72^ Skin biopsies from humans and mice with severe burns have demonstrated upregulation of NLRP3, IL-1β and IL-18, and increased cleaved caspase-1.^73^ These findings are further supported by the use of NLRP3 knockout and the NLRP3 inhibitor glyburide in a murine burns model, both of which led to a decrease in serum IL-1β, IL-18 and IL-6 concentrations.^73^ Additionally, Roth *et al* demonstrated that the AIM2 inflammasome is responsible for post-burn injury-induced immunosuppression in both mice and humans.^74^

Major trauma leads to inflammasome activation via tissue injury and resultant DAMP release, with up to 25% of patients with polytrauma developing ARDS.^75^ The neutrophils of patients with major trauma become primed with the subsequent propagation of systemic inflammation.^76^ Experimental models of severe traumatic brain injury (TBI) demonstrate neutrophil inflammasome activation, with a reduction in harmful inflammation seen following neutrophil depletion.^77 78^ Lin *et al* were the first to characterise the role of NLRP3 in a rodent model of severe TBI, with increased ASC and caspase-1 expression, NLRP3 inflammasome assembly, and release of IL-1β and IL-18 observed.^79^ These findings have been replicated in humans, with raised concentrations of NLRP3, IL-1β, ASC and caspase-1 in serum, cerebral spinal fluid and the cerebral cortex tissue of patients with TBI associated with increased injury severity and worsened clinical outcomes.^77 78 80^ The NLRC4 inflammasome has also been implicated in the inflammatory response to intracranial haemorrhage, and both NLRC4 and AIM2 inflammasomes may contribute to the sterile inflammation observed during cerebral and hepatic ischaemia-reperfusion injury.^81^,^83^

Transfusion-induced lung injury (TRALI) describes a life-threatening complication of massive blood transfusions that results in ARDS.^84^ TRALI is driven by aberrant neutrophil function through a two-hit process; the first hit is the priming of neutrophils, which may be due to a range of insults, leading to chemotaxis of neutrophils into the lung. Blood transfusion then acts as a second hit, whereby antibodies and bioactive lipids from red blood cells within the transfusion activate neutrophils.^84^ Stored blood products contain a range of DAMPS capable of both inflammasome priming and activation, with aged red blood cells known to activate the NLRP3 inflammasome.^85^

### Inflammasome modulation in ARDS

The inflammasome’s central role in ARDS makes it an attractive target for therapeutic modulation. To date, this approach has had limited success, likely due, at least in part, to an underappreciation of neutrophil inflammasomes’ central role in the pathophysiology of clinical ARDS.

#### Inflammasome blockade

Few inflammasome antagonists are available for human use, leading to limited clinical evidence of their effectiveness. Nevertheless, several treatments are under development and have shown promise in murine models of lung injury.^86^

MCC950 is a diarylsulfonylurea-containing compound that directly inhibits the NLRP3 inflammasome by binding to its NACHT domain, locking NLRP3 in an inactive conformation and thereby preventing ATP hydrolysis.^87^ Using a murine model of LPS-induced lung injury, Wang *et al* demonstrated that MCC950 inhibited pulmonary leucocyte infiltration and reduced mRNA and protein expression of IL-1β, IL-18 and caspase-1.^87^

GDC-2394 is another NLRP3 inhibitor that was investigated in NLRP3-associated disease.^88^ Despite a favourable pharmacokinetic and pharmacodynamic profile, its safety profile, including drug-related hepatic toxicity, precluded its development.^88^ The observed hepatotoxicity is not thought to be caused by NLRP3 inhibition, as several alternative NLRP3 inhibitors currently in development have not shown this effect.

A variety of other NLPR3 inhibitors are currently in development. RG6418 and MCC7840 are small molecule inhibitors with a structure similar to MCC950 that have undergone phase I trials for the treatment of the NLRP3-mediated disease cryopyrin-associated periodic syndrome.^89^ NT-0796 is an isopropyl ester that has shown promise in phase IIa clinical trials in Parkinson’s and cardiovascular disease.^90^ Other indirect methods of blocking NLRP3 involve inhibiting the protein-protein interactions essential for NLRP3 signalling. One example is HT-6184, a NIMA-related kinase-7 inhibitor, which recently completed phase I trials.^86^ No effective inhibitors of NLRC4 or AIM2 have yet been developed, but their increasingly recognised role in the pathophysiology of ARDS makes this an important area for further research.

It is important to acknowledge that inflammasome-independent pathways for IL-1β secretion exist, indicating that solely inhibiting the inflammasome may not be adequate to suppress IL-1β release and its subsequent inflammatory effects fully.^12 39^ Additionally, most inflammasome research on lung injury has been conducted in rodent models. Given the significant differences between the human immune system and that of other species, the applicability of these findings to humans may be limited.^35^

Caspase-1 inhibitors have been explored in vitro with mixed results and no successful translation into clinical practice.^12 14 32^ This may be due to the transient nature of caspase-1 activity, whereby following binding of pro-caspase-1 to the ASC speck, it then auto-cleaves to the active p33/10 isomer, rapidly followed by auto-cleavage to the inactive p20/10 form.^32^ This brief activity window provides limited opportunity for caspase-1 inhibition. Recently, strategies to neutralise the formation of ASC specks, thereby inhibiting ASC-dependent caspase-1 activation, have been explored. These drugs remain in early-stage development but include ASC-targeting antibodies (eg, IC100 and VHH_ASC_nanobody) and small molecule inhibitors (MM01).^91 92^

GSDMD inhibition is an attractive therapeutic target, given its role as a common output for all inflammasomes and its position upstream of IL-1β secretion.^93^ Disulfiram, necrosulfonamide and dimethyl fumarate inhibit oligomerisation and insertion of N-GSDMD into the cell membrane and have been found to suppress the inflammatory response and improve survival in murine sepsis models.^93 94^ Extension of these findings into humans has been limited by concerns about the blockade of GSDMD’s potential antimicrobial benefits and the finding that GSDMD knockout worsens renal and pulmonary pathology in murine inflammatory disease models.^94^ Dimethyl fumarate was investigated in the context of COVID-19 as part of the RECOVERY trial, but was not found to be beneficial.^95^ This lack of therapeutic efficacy may be explained by the hypothesis that human neutrophils do not undergo pyroptosis and that GSDMD is dispensable for IL-1β and IL-18 release. Thus, the benefits of GSDMD inhibition in neutrophil-driven diseases such as ARDS may be limited.^34^

β-hydroxybutyrate (BHB), a ketone body produced during periods of starvation, is an endogenous inflammasome antagonist.^96^ In vitro models have demonstrated BHB’s ability to inhibit NLRP3 inflammasome activation in human neutrophils and macrophages, alongside modulation of harmful downstream functions such as NETosis.^96^,^99^ McNelly *et al* have shown ketogenic feeding to be safe and feasible in critically ill patients, and it thus represents a potential avenue for modulating inflammasome-driven inflammation in critical illness.^100^

#### Cytokine blockade

IL-1β is a key inflammasome output and a known driver of ARDS.^10^ Clinical blockade of IL-1β has been achieved using IL-1 receptor antagonists (eg, Anakinra), monoclonal antibodies (eg, Canakinumab) and recombinant proteins (eg, Rilonacept).^17 25^ To date, the success of IL-1β blockade in critical illness has been limited, with benefits only seen in a subgroup of hyperinflammatory patients.^9 17^ Anakinra blocks both IL-1α and IL-1β and has been used in rheumatological diseases for many years, with its use in critical illness growing.^101^ A randomised control trial by Fisher *et al* investigated its use in patients with sepsis and found no benefit. However, post-hoc re-analysis of the data suggested benefit in patients with a hyperinflammatory phenotype and the greatest predicted mortality.^102^ As 30% of patients with ARDS present with a hyperinflammatory phenotype, this may represent a cohort of patients that would benefit from IL-1β blockade. Furthermore, Engeroff *et al* investigated IL-1 blockade in a murine model of LPS-induced acute lung injury and found Anakinra to significantly reduce harmful inflammation.^103^

Anakinra has been investigated in COVID-19-associated respiratory failure in humans, with contrasting results. When used early in the disease process, a signal towards mortality benefit and a reduction in markers of an excess inflammatory response was seen in some patients with ARDS.^17 104^ However, concerns regarding the use of Anakinra in COVID-19 were raised in randomised control trials, citing an increased incidence of secondary infections, transaminitis and neutropaenia.^105 106^ These contrasting findings highlight the need for further research to understand inflammasome activation dynamics in this context.^48^ While IL-1β blockade is, in principle, a promising therapeutic approach, current limitations to its use include concerns over its safety in critical illness and the absence of effects on other inflammasome outputs (ie, IL-18) or downstream effects (ie, pyroptosis and NETosis).

IL-18 is another inflammasome output associated with ARDS severity and mortality, but little work has been undertaken regarding its therapeutic blockade in this context.^106^ GSK1070806 was tested to enhance graft function after renal transplant but proved ineffective. It is currently being studied in the area of Crohn’s disease and atopic dermatitis.^107^ The dual anti-IL-1β/IL-18 monoclonal antibody MAS825 was studied in COVID-19, reducing inflammatory markers (eg, IL-6) but showing no clinical benefit, and is now being trialled in *NLRC4*-mutation associated macrophage activation syndrome.^108^ Studies investigating the blockade of IL-18 via its endogenous inhibitor, IL-18 binding protein, remain in the early stages.^109^

### What is next for inflammasome research in ARDS?

Inflammasome activation and IL-1β secretion are key drivers of ARDS and represent attractive therapeutic targets, yet the investigation of their modulation in humans has, to date, yielded mixed results. This is likely because despite being the dominant cell type in the pathophysiology of ARDS, the pathways through which human neutrophils secrete IL-1β are incompletely defined. Furthermore, the NLRP3 inflammasome dominates the current literature, which may be due to its pivotal role in ARDS, but it could also reflect the relative paucity of research investigating alternative inflammasomes in relevant human cells. The extent of the involvement of non-NLRP3 pathways is an important unanswered scientific area. Given the differences between mammalian species and cell types, future work should concentrate on the full range of human cells.

### Conclusion

ARDS remains a major cause of death in critically ill patients. The inflammasome plays a central role in the pathophysiology of ARDS yet remains poorly defined in human neutrophils. An improved understanding of these pathways represents a little-explored route for developing potentially beneficial therapies.

## Supplementary material

10.1136/thorax-2024-222596 online supplemental file 1
