# Supplementary material for: Role of inflammasomes in acute respiratory distress syndrome
Source: Thorax. 2025 Jan 30;80(4):e222596. doi: 10.1136/thorax-2024-222596 (PMC12015084; doi:10.1136/thorax-2024-222596)
Supplement: online supplemental file 1 [file thorax-80-4-s001.pptx]

## Slide 1
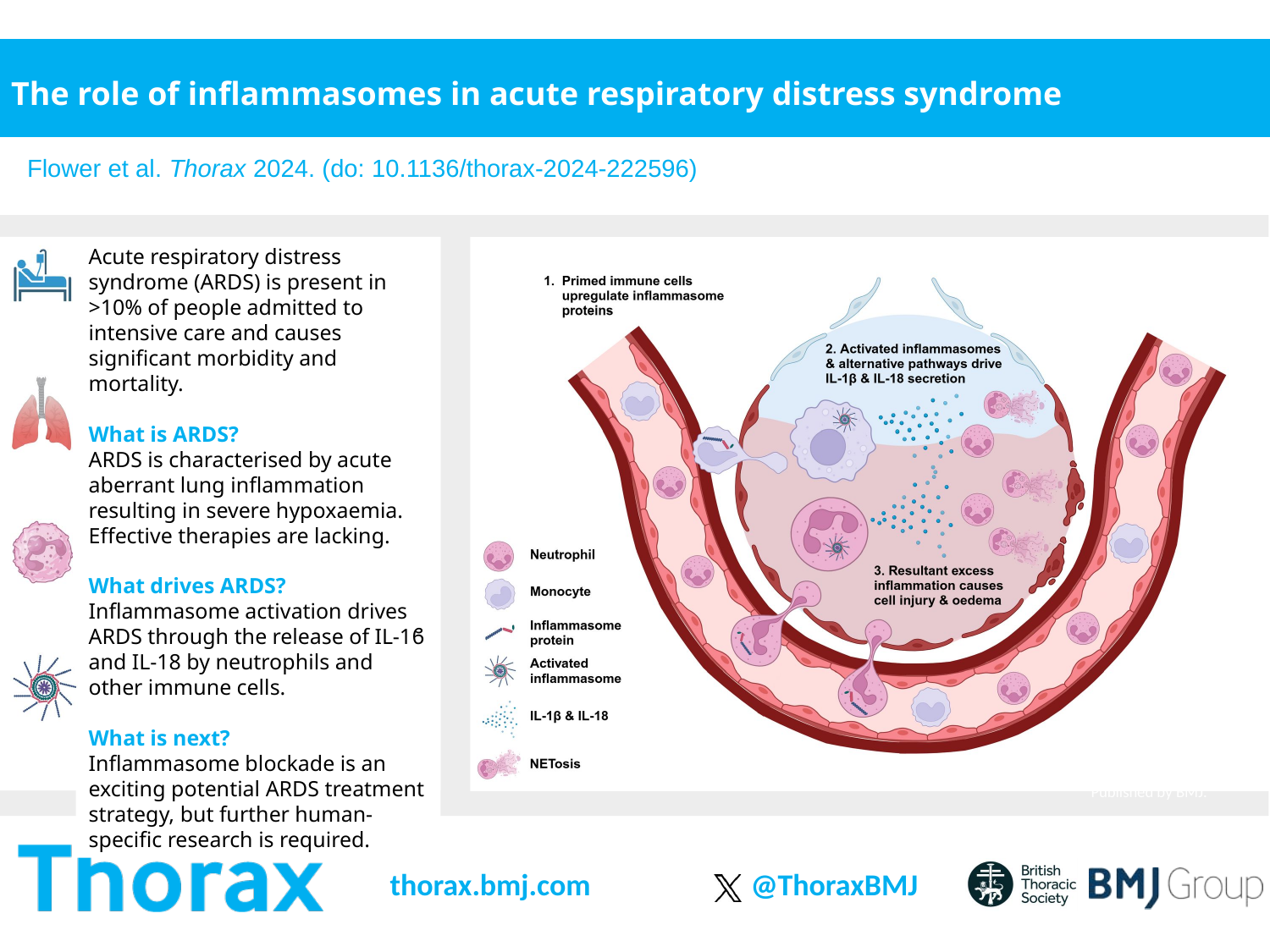

The role of inflammasomes in acute respiratory distress syndrome
Flower et al. Thorax 2024. (do: 10.1136/thorax-2024-222596)
Acute respiratory distress syndrome (ARDS) is present in >10% of people admitted to intensive care and causes significant morbidity and mortality.
What is ARDS?
ARDS is characterised by acute aberrant lung inflammation resulting in severe hypoxaemia. Effective therapies are lacking.
What drives ARDS?
Inflammasome activation drives ARDS through the release of IL-1ϐ and IL-18 by neutrophils and other immune cells.
What is next?
Inflammasome blockade is an exciting potential ARDS treatment strategy, but further human-specific research is required.
© Author(s) (or their employer(s) 2019. Re-use permitted under CC BY. Published by BMJ.
thorax.bmj.com @ThoraxBMJ
